# 3D Printing at Micro-Level: Laser-Induced Forward Transfer and Two-Photon Polymerization

**DOI:** 10.3390/polym13132034

**Published:** 2021-06-22

**Authors:** Muhammad Arif Mahmood, Andrei C. Popescu

**Affiliations:** 1 Laser Department, National Institute for Laser, Plasma and Radiation Physics (INFLPR), 077125 Magurele, Ilfov, Romania; arif.mahmood@inflpr.ro; 2 Faculty of Physics, University of Bucharest, 077125 Magurele, Ilfov, Romania; 3 Center for Advanced Laser Technologies (CETAL), National Institute for Laser, Plasma and Radiation Physics (INFLPR), 077125 Magurele, Ilfov, Romania

**Keywords:** micro-printing, laser-induced forward transfer, two-photon polymerization, 3D printed materials and their properties

## Abstract

Laser-induced forward transfer (LIFT) and two-photon polymerization (TPP) have proven their abilities to produce 3D complex microstructures at an extraordinary level of sophistication. Indeed, LIFT and TPP have supported the vision of providing a whole functional laboratory at a scale that can fit in the palm of a hand. This is only possible due to the developments in manufacturing at micro- and nano-scales. In a short time, LIFT and TPP have gained popularity, from being a microfabrication innovation utilized by laser experts to become a valuable instrument in the hands of researchers and technologists performing in various research and development areas, such as electronics, medicine, and micro-fluidics. In comparison with conventional micro-manufacturing methods, LIFT and TPP can produce exceptional 3D components. To gain benefits from LIFT and TPP, in-detail comprehension of the process and the manufactured parts’ mechanical–chemical characteristics is required. This review article discusses the 3D printing perspectives by LIFT and TPP. In the case of the LIFT technique, the principle, classification of derivative methods, the importance of flyer velocity and shock wave formation, printed materials, and their properties, as well as various applications, have been discussed. For TPP, involved mechanisms, the difference between TPP and single-photon polymerization, proximity effect, printing resolution, printed material properties, and different applications have been analyzed. Besides this, future research directions for the 3D printing community are reviewed and summarized.

## 1. Introduction

Recently, various fields have adopted three-dimensional (3D) microfabrication, including photonics, microfluidics, and tissue engineering. In these areas, part fabrication requires fast prototyping with arbitrary and multifaceted geometries. The manufacturing technology selected for this purpose will allow to choose a wide diversity of materials to synthesize them at a scale within the range of nanometers and centimeters. During the last two decades, various research groups have made tremendous efforts to develop and improve the direct-writing technologies capable of manufacturing micro-3D objects with high reliability and nano-level pixels [1]. The broad field of writing methods focused on energy beams and ink flows, where parts are manufactured by the layer-after-layer method, can be used to investigate different cases of such improvements [2,3].

In the manufacturing of 3D microstructures, direct laser writing (DLW) methods are gaining attention day-by-day. The laser beam wavelength and operating modes (continuous or pulse) can be utilized for a wider range of physio-chemical methods. Furthermore, laser manufacturing is more consistent, cost-effective, and simple to use than traditional manufacturing methods, thus reducing the obstacles to their use in science and commerce [4]. There are various direct laser writing techniques given in Table 1.

In DLW, two- and three-dimensional structures are manufactured by guiding a laser beam in an anticipated outline. DLW shows various characteristics required by multiple electronics and medical devices production. One of the advantages of DLW methods over conventional manufacturing methods is that intricate geometries can easily be manufactured to encounter the requirements of a specific application. The following steps are followed in every DLW process [11]:

- A three-dimensional model is generated using CAD software.
- This CAD model is converted generally into “.STL” format.
- Various layers having thin cross-sections are produced from step (ii). This step is also known as slicing.
- The 3D part is manufactured via computerized numerical control (CNC) codes attained from step (iii).

Recently, LIFT and TPP have gained popularity for the printing of 3D shapes at micro-level, a feature demanded by medical, electrical, and sensing devices. To the best of our knowledge, various research studies have been carried out on LIFT and TPP. To highlight the need and significance of these two techniques, no author has tried to compile the components and their properties printed by LIFT and TPP having a wide range of materials such as polymers, metals, and nanomaterials. In this novel review article, a detailed discussion has been carried out on LIFT and TPP. Section 2 discusses the schematic of LIFT, various LIFT methods, flyer velocity and shock wave formation, materials printed by LIFT and their properties, and applications of LIFT-ed materials. Section 3 presents a discussion on TPP schematic, the mechanism behind TPP, the difference between TPP and single-photon polymerization, proximity effect, printing resolution, TPP-ed materials properties, and applications of materials printed by TPP. Conclusions and future work are discussed in the Section 4.

## 2. Laser-Induced Forward Transfer (LIFT)

**Figure 1:**
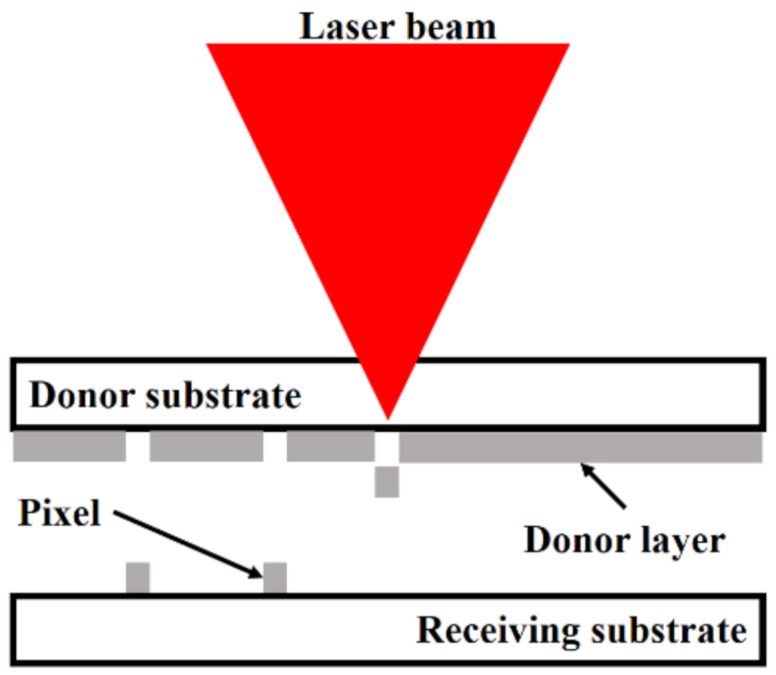
Schematic of LIFT technique.

LIFT is a printing method that allows material deposition in a tiny quantity, resulting in a solid or liquid pixel with high resolution. For the very first time, Bohandy et al. [12] developed and explained this technique. Since then, various techniques based on LIFT have been established for multiple materials printings used in specific applications. Figure 1 presents a schematic of the processes involved in the LIFT technique [13,14,15]. As presented in Figure 1, the solid-phase pixels are transferred after irradiating the thin layer or film (donor) via a laser-pulse, made up of an engrossing material placed on a translucent substrate. As the layer’s irradiation occurs, the light to matter encounter happens at the periphery, generating a stout pressure locally. Due to this phenomenon, a minor portion of the donor film (pixel) is evicted and accumulated on the receiving substrate, located close to the donor [16].

An incident laser beam’s diameter and profile usually control the magnitude and shape of the evicted substance. Structures having typical sizes of a few micro-meters can be printed by the LIFT technique. In the liquid stage of droplet printing, the donor layer consists of a liquid, viscous layer having a thickness of a few micro-meters. The laser attacks the liquid layer directly if the layer’s absorbance is sufficient to ignite the vapour bubble formation. This phenomenon pulls the fluid encircling the bubble, forming an extremely slim and steady jet, which spreads up to the exterior and makes a drop [17]. Table 2 compiles the various LIFT-printed materials.

LIFT is a single action DLW technique, which can deposit the localized micro-patterns of almost every material. Sensitive materials can be printed without changing their properties. This also allows direct printing of multilayers in a solvent-free atmosphere, deprived of requiring any sort of shadow mask or vacuum system [35,36]. This method has recently become famous as an alternative method to manufacture electronic components [37], organic transistors [38,39,40], organic diodes for light-emitting [41,42,43], micro-electro-mechanical systems [44,45], sensing devices [46,47,48,49], and biological tissue [50,51]. LIFT has a few pros and cons. For this purpose, the efficient expansion of a laser printing method necessitates a comprehensive understanding of the physio-chemical phenomenon causing the material’s ejection and accumulation.

### 2.1. Various LIFT Methods

LIFT is classified into the following methods, based on the nature of the material to be transferred, explained in Table 3.

### 2.2. LIFT: Flyer Velocity and Shock-Wave Formation

**Figure 2:**
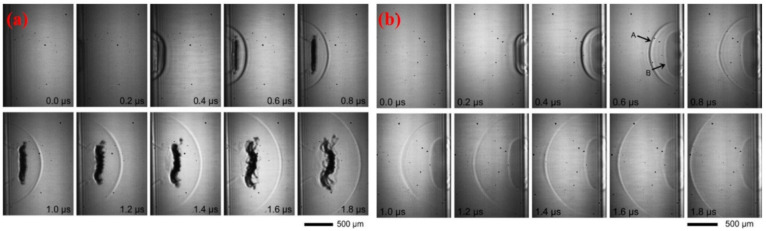
(**a**) Backside images captured for 460 nm triazene-polymer ablation at laser fluence equal to 110 mJ·cm^−2^, and (**b**) front view ablation of 460 nm triazene-polymer ablation at laser fluence equal to 110 mJ·cm^−2^. In both images, laser is at the left-side, and the substrate is placed at right-side [57]; with permission from Elsevier.

The transfer of pixels is usually encouraged by mechanical forces due to laser beam energy absorption at the boundary of the translucent donor (substrate) and receiver (film). A rapid upsurge in temperature intensity and pressure happens within the limited volume, which disintegrates the donor around the irradiated region, thus pushing the pixel away to the receiver. Besides this, a shock-wave is generated, spreading in the pixel-front. Figure 2a shows the images captured, in the case of triazene-polymer (=460 nm), irradiated with a laser having fluence equal to 110 mJ·cm^−2^. The images were captured after rotating the sample up to 180° to have a clear front view of ablation, as shown in Figure 2b [57]. A noticeable shock-wave with the same profile and extension characteristics can be analyzed. The primary difference is the product front’s replacement with an opaque entity evicted from the substrate such that the flyer becomes noticeable after 0.4 ms irradiation time. Initially, the flyer has a precise flat shape consistent with the ablated spot. Later on, it enlarges, becomes inaccurate, and loses its original profile. The flyer existence shows that a single laser pulse does not ablate the entire triazene layer at the selected fluence. The flyer contains an undecomposed polymer, evicted by the thrust pressure made throughout ablation and breakdown of the beneath part.

The pixels’ ejection velocity is determined from the shadowgraph’s analyses, which primarily depends on laser beam fluence and donor thermo-physical characteristics. When the pulse duration is in the range of a picoseconds~microseconds, no substantial alteration in velocity is identified. The solid pixels are usually expelled with velocities in the range of 200–1200 ms^−1^. Besides, it is also probable to emit pixels at a very small velocity equal to 34 ms^−1^, resulting in no shock-waves formation. Such conditions were tested while transferring lead zirconate titanate (PZT) using a laser of 100 fs [58]. Apart from the laser energy captivated by the substance to heat the substrate, another portion is utilized to fracture the substrate and moved away to the receiver. For the LIFT process, the perfect conditions are defined as:

(a) Bound heat-diffusion within the substrate to dodge the impairment and to generate the molten fragments.
(b) A significant portion of laser energy used to fracture the substrate and hold the pixel speed for a short time interval to confirm the pixel’s flat arrival at the receiver.
(c) In the case of particular materials, the femto-second (fs)-laser permits the regulation of this energy distribution.

**Figure 3:**
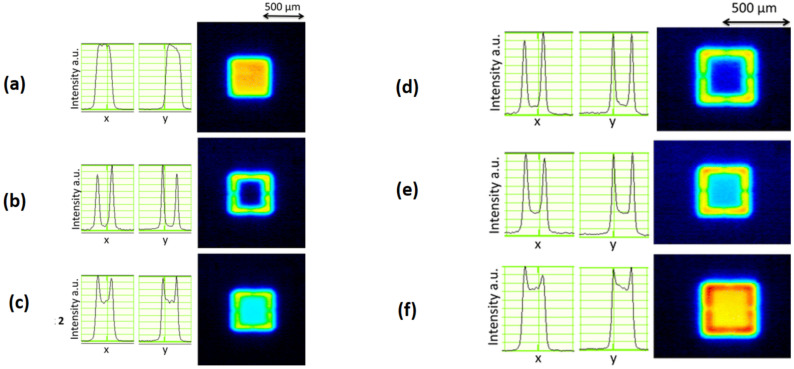
Beam profiles with associated intensities along the *x*- and *y*-directions in the LIFT experimental setup at the donor layer: (**a**) a square mask, (**b**) with mask at contours, (**c**) with a combination of (**a**) and (**b**) techniques, (**d**) high-intensity ratio, (**e**) medium intensity ratio, and (**f**) low-intensity ratio [61]; with permission from Elsevier.

In the solid phase, the generation of shock-wave is the principal disadvantage of LIFT. Fardel et al. [59] confirmed that, when it arrives at the donor, back-scattered, an interaction occurs between the reflected shock-wave and pixel. This interaction stops the pixel motion and causes pixel’s de-shape. Using a gap of a few micrometers between the donor and receiver, laser printing can be achieved at atmospheric pressure. Various printing experiments were performed under vacuum to exclude this shockwave, but the flyer broke due to its very high velocity when arriving at the substrate [59]. Therefore, a compromise has to be made between the intensity of shock-wave formation and the pixel’s velocity by controlling the gas pressure and laser beam fluence [60]. Usually, the working pressure is within the range of 10–100 mbar, allowing the pixels’ safe travel with a gap of less than 50 mm. This process involves the mechanical force formation that breaks the donor. So, printing a brittle material or thick layer (>a few micrometers) is a challenging task. There are two methods used to solve this issue. Rapp et al. [61] utilized a particular laser profile to independently regulate the energy needed to break a film, focused at the beam’s edge. It can be observed in Figure 3 that, after integrating the two beams having different energies and shapes, the laser fluence can be enhanced at the beam’s edge, while a low fluence value can be obtained in the central beam portion. This technique allows printing polymer films with more than 1 mm thickness without compromising resolution [62].

### 2.3. Various Materials Printed by LIFT and Their Properties

**Figure 4:**
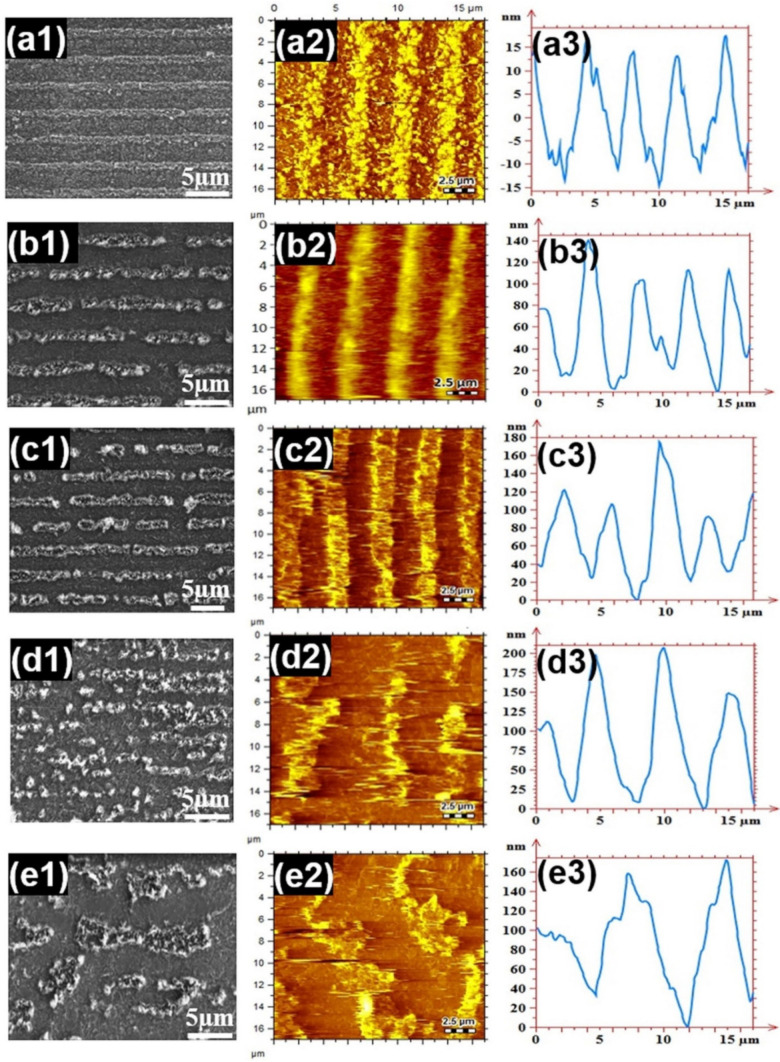
Laser fluence and stripes morphologies: (**a1**–**e1**) scanning electron microscopy, (**a2**–**e2**) atomic force microscopy, and (**a3**–**e3**) stripes height profiles variations; [63]; with permission from Elsevier.

Shen et al. [63] used LIFT to transfer micro-stripes of polyimide, with a thickness of 1.2 µm, with laser fluence of 39 mJ·cm^−2^ and 50 pulses. The deposited strip height (20 to 140 nm) increases with an increment in the laser fluence (34–52 mJ·cm^−2^). Figure 4a–e show the influence of the laser beam fluence, having nano-second laser pulse, on the polyimide donor-film (thickness ~1.2 µm). The laser fluence was used in the range of 34~51 mJ·cm^−2^, while the laser pulses were forty. From Figure 4(a1–e1), one can observe the scanning electron microscopy (SEM) images of stripes passed on via LIFT. At relatively low fluence (34 mJ·cm^−2^), the stripes showed an entire structure with sharp contours, as shown in Figure 4(a1). On the other hand, the stripes began to break-down at the laser fluence higher than 34 mJ·cm^−2^ and balling phenomena can be analyzed clearly in Figure 4(b1–e1). In Figure 4(a1–c1), the size of micro-stripe is 3 µm. Besides, the stripes morphologies were examined using atomic-force microscopy (AFM). These results are presented in Figure 4(a2–e2). The height profiles of the stripes are shown in Figure 4(a3–e3). One can analyze that an increment in the laser fluence causes non-uniformities in the stripe height.

**Figure 5:**
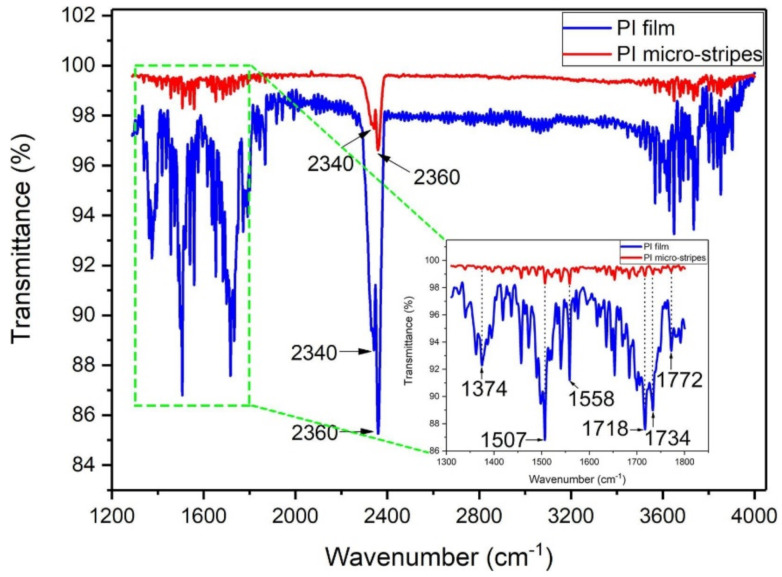
Fourier transform infrared spectroscopy of polyimide and stripes formation [63]; with permission from Elsevier.

To identify the polyimide stripes composition, Fourier transform infrared spectroscopy (FT-IR) measurement was performed. Figure 5 displays the results of FT-IR spectra. The results show the spectra of polyimide and stripes generated after LIFT. It is worth mentioning that only a material fraction was ejected from the polyimide-donor film. Hence, the polyimide films absorption intensity was higher than stripes. In Figure 5, polyimide showed three absorption bands at (a) 1374 cm^−1^, (b) 1734 cm^−1^, and (c) 1772 cm^−1^, which correspond to the chemical bond vibration between Carbon and nitrogen, and carbon and oxygen (symmetric and asymmetric). At 2340 cm^−1^ and 2360 cm^−1^, the bands are allocated to carbon dioxide captivation, produced during acrylic acid imidization. Finally, the FT-IR spectrum shows that the stripe’ composition remains the same when transferred from polyimide substrate [63].

**Figure 6:**
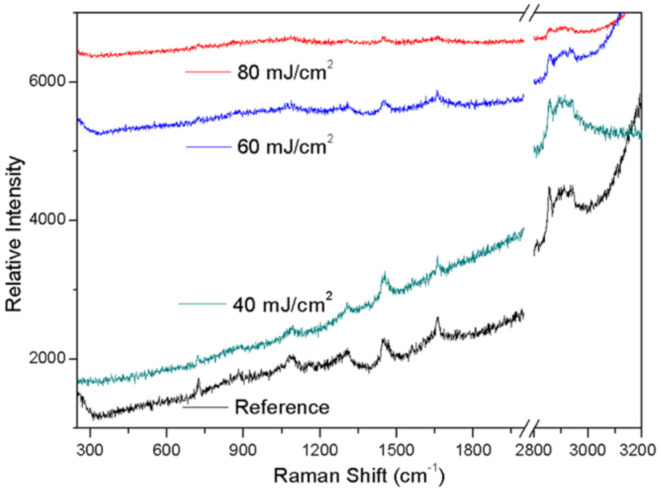
Liposome Raman spectrum [64]; with permission from Springer.

Papavlu et al. [64] demonstrated the capability of LIFT for the pattering of liposomes at a micro-scale. These patterns are commonly used in biosensors and drug delivery systems. The solution was optimized by using dynamic release layers with various thicknesses. A clean transfer was attained for triazene polymer film having thickness more than 150 nm. Different distinct outlines were obtained with the laser beam fluences in between 40–60 mJ·cm^−2^ and 193 nm irradiation. They determined that the distance between target and substrate does not influence the micropatterns morphologies. Figure 6 shows the Raman spectroscopy of the printed samples. Moreover, Raman spectroscopy for the chemical composition of liposomes remained the same for the laser fluences between 40 and 60 mJ·cm^−2^. Liposome arrays were manufactured with the least volume, suggesting that LIFT is a promising practice for liposome patterning.

**Figure 7:**
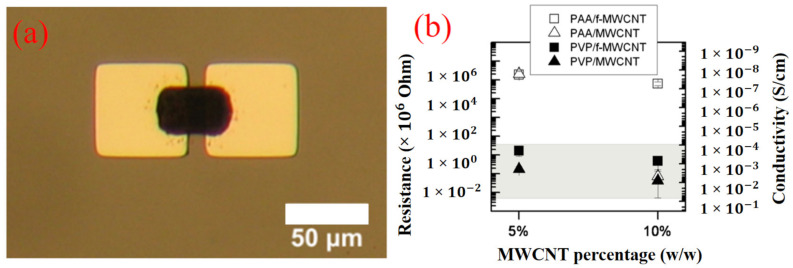
LIFT depositions: (**a**) Optical microscopy image of polyvinylpyrrolidone (PVP) + functionalized multiwall carbon nanotubes (f-MWCNT), and (**b**) electrical characteristics [65]; with permission from Applied Physics Letters.

Boutopoulos et al. [65] suggested that LIFT is an alternate and up-coming method for the composite layers printing of polymers + carbon nanotubes (CNT). LIFT deposited polyvinylpyrrolidone (5%) + functionalized multiwall carbon nanotubes and polyacrylic acid (10%) + functionalized multiwall CNTs layers showed a uniform CNTs distribution within polymer matrices. The composite layers’ electrical characterization was accomplished by accumulating pixels from all the targets onto aluminum micro-electrodes detached by a breach of 10 µm. Figure 7a shows a precise 30 × 40 µm^2^ polyvinylpyrrolidone (5%) + functionalized multiwall CNTs pixel printed on aluminum micro-electrodes using laser fluence of 220 mJ·cm^−2^. A Hewlett Pack was used to characterize all the deposited layers. Figure 7b shows the deposited composite layers’ confrontation and electrical-conduction using direct-current. It can also be analyzed that, for a given composition of polymer and CNTs, composite layers with functionalized multiwall CNTs methodically presented low conduction related to non-functionalized multiwall CNTs. This can be attributed to the functionalized CNT’s primarily feebler electrical characteristics compared to non-functionalized CNTs.

Dinca et al. [66] presented LIFT depositions for both single- and multiple-pixel depositions. The final goal was to obtain polyethyleneimine (PEI) and polyisobutylene (PIB) continuous pixels on surface acoustic waves (SAW) surfaces. After depositions with multiple fluences, the coated transducers’ responses were measured. They determined that a laser fluence below 625 mJ·cm^−2^ was obligatory to stop interdigital transducers impairment for the sensor devices. These measurements show that LIFT can print sensitive polymer pixels onto the transducer devices. Feinaeugle et al. [67] demonstrated a thermo-electric energy harvesting device’s fabrication via LIFT of intact solid thin films. Thermo-electric materials, including bismuth telluride (Bi_2_Te_3_), bismuth selenide (Bi_2_Se_3_), and bismuth antimony telluride (Bi_0.5_Sb_1.5_Te_3_) were printed via an excimer laser (nanosecond pulse) on an elastomeric polydimethylsiloxane coated glass substrate. The generator Seebeck coefficient and series resistance were 0.17 mV·K^−1^ and 10 kΩ, respectively. Stewart et al. [68] used a UV-absorbing triazene polymer (TP) as a DRL layer to propel other materials forward without any damage. A picosecond pulse length was used to irradiate TP-films, which produced considerable deviations in the ablation process compared to the nanosecond pulse ablation. The shorter pulse length declines the ablation threshold and ablation threshold, observed in thinner films less than 100 nm. This propels the shockwave and flyer faster and forms a more disjointed flyer. These effects are due to the picosecond laser’s pulse length timescale, resulting in higher energy ablation products by elevating the TP’s effective absorption. This, in return, reduces losses due to the fast process.

Karnakis et al. [69] used a KrF excimer laser with wavelength (248 nm) and pulse duration (30 ns). Pyrene-doped PMMA donor film was irradiated to transfer pyrene molecules to poly-butyl-methacrylate (PBMA) substrate. The laser pulse passed through a beam homogenizer to ensure the same energy distribution on the whole area of the laser spot and was focused on a 1.2 cm spot size. Both films were spin-coated on quartz substrates. The donor film had a thickness of 0.1–4 µm thickness, while the receiving polymer was 2 mm thick. It was found that the receiving polymer surface fluorescence spectra will be changed if the molecules decayed or just stuck to the polymer surface. However, the receiver surface reserved its original smoothness. Nakata et al. [70] examined the LIFT of Rhodamine 610 laser dye on a SiO_2_ substrate via Nd: YAG laser (532 nm and 8 ns). Ethanol solution droplets (donor film) were applied on a gold (base film), placed at 15 µm from the SiO_2_ substrate. The fluorescence emission was optimal at laser fluence (49 mJ/cm^2^) under vacuum condition.

**Figure 8:**
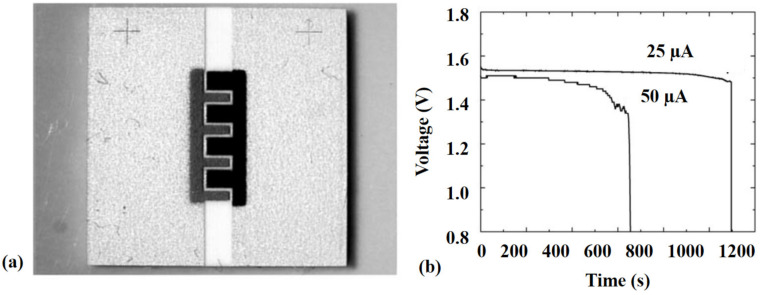
(**a**) Optical micrograph of Ag_2_O/Zn alkaline micro-battery by LIFT, and (**b**) discharging behavior of alkaline micro-batteries at 25 and 50 µg as a time function [73]; with permission from Springer.

Pentacene (PE) is ideal for replacing amorphous silicon in organic thin-film transistors (TFTs). Blanchet et al. [40] presented experimental analyses of thin PE-film deposition via Nd-YAG laser (266 nm and 10 ns). The laser beam was collimated onto an area of 3 cm^2^ target surface under an ultrahigh vacuum of 10^−7^ Torr. The laser-evaporated PE-films’ semiconducting performance was analyzed. It was found comparable with the PE-thin films deposited by thermal evaporation. Polyaniline (POLY) is a unique conducting polymer. Blanchet et al. [71] carried out a pixelized transfer of POLY + nanotube structures via the thermal imaging technique. An infrared laser (40 W and 780 nm) was engrossed over a thin metallic-layer (donor base) having POLY coating. Light to heat conversion resulted in gaseous products and shockwaves, resulting in the material transfer from the substrate to the receiver. Using this method, the composite’s wide line equal to 500 µm was printed with a 7 µm gap with a 2 S/cm conductivity. Blanchet et al. [72] printed a TFT backplane having 5000 transistors with 20 µm channel on a substrate (50 × 80 cm^2^). Due to the deficiency of built-in registration, the sources and drains were shifted from the gate’s center. The maximum misregistration was less than 200 µm. Electronic systems printed using this technique offer low-cost, higher mechanical flexibility, and large area coverage than traditional silicon technologies. Pique et al. [73] applied the LIFT technique to manufacture rechargeable Li-ion micro-batteries via Nd: YVO_4_ laser (355 nm). The produced micro-battery and corresponding discharging behaviour are provided in Figure 8.

### 2.4. Applications of LIFT: Organics, Electronics, and Sensors

LIFT is a novel and unique technique alternative to incumbent techniques, e.g., inkjet printing, due to its capability to deposit pico-litre-sized droplets [74]. This is beneficial for biological applications due to the use of a small quantity of reagent and a wide range of rheological properties. Besides, it can be executed in a simple lab environment. Using this method, specific and moderate depositions of complex molecules and eukaryotic cells show the exclusive capabilities of this technology. LIFT has a potential to print liposomes [75], deoxyribonucleic acid [18,22,76], and proteins [77] with high resolution. Tissue printing is one of the latest applications of LIFT, proposed to replace burnt-cells [78,79]. These printed tissues behave like human osteosarcoma, endothelial and osteoprogenitor cells [77,80,81] that have been successively printed. In-vivo and in-vitro testing was carried out on mice and showed excellent results [82].

This technique is used to heat and light-sensitive materials, which cannot be irradiated by the pulsed-laser. For this purpose, organic light-emitting diodes (LEDs) [83] and thin-film transistors (TFTs) [78] have been printed by this method using different materials such as semiconductors and silver inks. Rapp et al. [84] achieved an organic-TFT device using the LIFT technique. Distyryl-quaterthiophene (DS4T) was deposited on a donor substrate and transported with pico-second laser pulses on Si/SiO_2_-based receiver substrate. The polymer layer showed high laser-absorption, and DS4T pixels printed on the receiver substrates presented excellent morphological properties. The LIFT technique manufactured polymeric LED via the decomposition of a thin triazene polymer film via a Xe-Cl excimer laser [43]. The deposition process allows for the transfer of a bi-layer containing electroluminescent enclosed with an aluminum electrode. The soft transfer resulted in well-resolved pixels (=500 µm). Stewart et al. fabricated small molecules of Alq_3_ organic-LED to produce RGB pixels [85]. A coating of tetra-butyl ammonium hydroxide was placed at the aluminium cathode top to enhance the electron injection in the Alq_3_, by more than 600%. The luminance and the efficiencies of these organic-LEDs were better than conventional LEDs in blue pixels.

LIFT has also been used to manufacture sensing devices. One of the drawbacks of LIFT is that it produces debris during material transfer, which is not beneficial for microelectronics. However, it is not a real problem for sensors manufacturing. On the other hand, LIFT provides an opportunity to print various sensitive materials to enhance the selection of the devices. In this context, Pietrantonio et al. [47] printed a sensor array (SA) based using LIFT with three polymers: (a) poly-epi-chlorohydrin, (b) poly-isobutylene, and (c) poly-ethylenimine. A Xe-Cl laser was used. The targets were coated by the MAPLE technique. SA was examined with exposure to dimethyl methyl phosphonate, dichloromethane, and ethyl acetate. The finest sensitivities for dimethyl methyl phosphonate and dichloromethane obtained using a poly-epi-chlorohydrin coated sensor were 66.23 Hz/ppm and 0.034 Hz/ppm. Furthermore, the best sensitivity for ethyl acetate was 0.33 Hz/ppm for poly-isobutylene. Mattle et al. carried out the transfer of SnO_2_ by LIFT for gas sensor applications. Various donor SnO_2_ substrates, with and without triazene polymer, were printed by LIFT. SnCl_2_(acac)_2_ based donor films were prepared and transferred using UV light. Transferred material conductivity was measured at 385 °C. The resistances of all successfully transferred SnO_2_ layers are in the range of 200 kΩ to 4 GΩ. Touloupakis et al. [86] used LIFT to transfer an electron from photosynthetic biomaterials immobilized on screen-printed electrodes. Thylakoid droplets with a current intensity of approximately 335 nA (28 ng), was selected. It was found that the efficiency of the photosynthetic system can be enhanced by the LIFT process (50–70%) to develop an efficient and more sensitive biosensor to detect herbicides at nano-molar concentrations. A few more transferred materials and their applications are summarized in Table 4.

## 3. Two-Photon Polymerization (TPP)

**Figure 9:**
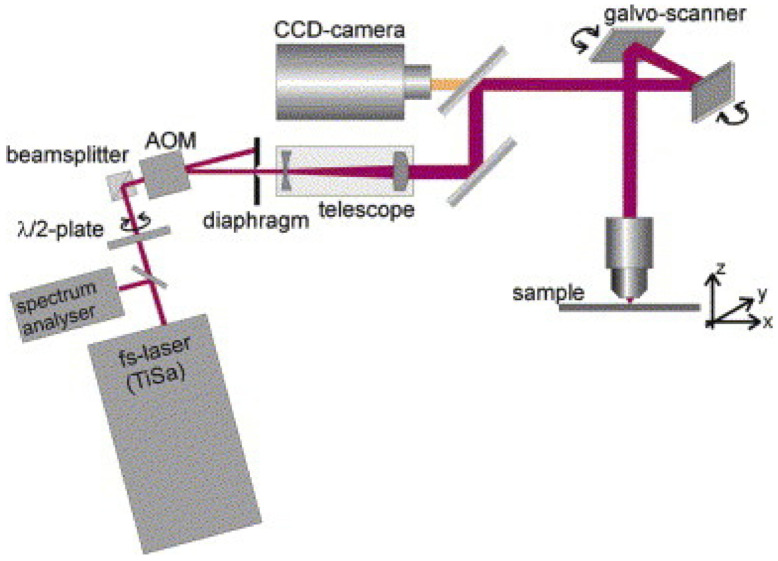
Two-photon polymerization setup schematic [102]; with permission from Elsevier.

Figure 9 shows the schematic of a typical TPP setup [102]. A femtosecond laser is used to achieve two-photon absorption. Microscopic objectives are applied for laser beam focusing [103]. The voxel size and polymerized structure’s feature primarily depend on the energy distribution, supervised by the focusing optics [104]. In TPP, either resin moves with respect to a fixed optical focal point, or the optical focal point moves with respect to resin. However, the resin movement w.r.t the laser is easily attained via Galvano mirrors (Figure 9). The scanning can be performed in x- and y-axes, while the piezoelectric stage translates the resin along the *z*-axis [105,106]. Moreover, higher printing resolution (1.20 nm/step) can be achieved using Galvano mirrors as compared to the piezoelectric stage with (0.99 nm) [105,106]. TPP is a valuable technique to manufacture 3D-structures having micro/sub-micron features. However, this process has limitations in terms of tolerance for functional parts caused by the submicronic size of components features.

### 3.1. Overview of TPP Mechanism and It’s Difference with Single-Photon Polymerization (SPP)

TPP is a photo-chemical process introduced by a femto-second laser firmly concentrated into the volume of the photosensitive resin using an optical objective. This process contains three sub-processes: (a) initiation, (b) propagation, and (c) termination [107]. In the first process, the monomer (in liquid state) usually reaches the excited state after absorbing two photons via non-linear mechanisms. The energy provided by the femto-second laser beam starts polymerization, and a solid material forms in the laser-irradiated area. TPP is fundamentally different from SPP. TPP is used to start the same molecular transition in the photo-initiator as those originated by SPP, but it is a weak and non-linear effect [108]. TPP happens when two photons excitation wavelengths are classically about twice the one-photon excitation wavelengths [109]. The possibility of two-photons concurrent absorption is very low. Hence, very high intensity of light (=TW/cm^2^) is needed to acquire considerable TPP [110,111]. So, the TPP process is restricted to an internal sub-diffraction volume by converging femto-second laser pulses to a diffusion-limited spot. By cautiously selecting the wavelength and absorption factor, one can assure that the material polymerizes via TPP instead of SPP. For instance, UV-curable resists for SPP at IR-wavelengths but strongly absorbs at half of the wavelength. Thus, TPP is carried out by choosing a near-IR laser (fs) and UV-curable resists. This grouping has been widely used to manufacture tens of micron characteristics via TPP [112,113,114,115]. It is worth mentioning that two-photon absorption is a crucial process for TPP.

### 3.2. Laser Damage in TPP: Proximity Effect

In TPP, laser impairment happens when the interacted laser intensity is much higher than the threshold intensity needed to resist polymerization [116]. It causes bubbles formation and hampers the additional printing process, thus failing the deposited structure. Before carrying out TPP, one should confirm that the laser power and printing speed are correctly designated to fall under the impairment gateway. It can be done after conducting various experiments by varying laser powers and scanning speeds [117]. The proximity effect rises mainly because of the variations in the resist absorption band due to the curing procedure. At near-IR wavelength, the polymerized resin absorptivity surges to a non-negligible value during curing. The proximity effect has the capability to lessen the laser impairment limit and produce a featured part with optimum density.

### 3.3. TPP Printing Resolution

In TPP, printing resolution is an essential variable expressing micro-/nano-structures feature dimensions. It is usually expressed function of the voxels and/or lines. For fabrication, two types of scanning methods have been identified: (a) pin-point scanning and (b) continuous scanning [118]. In both scanning modes, the TPP resolution is expressed as the voxels size and lines width. Usually, the voxel shows the resemblance to a spinning ellipsoid having smaller lateral diameters than the axial length. The axial length to oblique diameter ratio is known as the aspect ratio [118]. Various factors influence the printing resolution discussed in Table 5.

### 3.4. Materials Printed by TPP and Their Properties

#### 3.4.1. Mechanical Characteristics and Bonding Strength

TPP-produced structures can be utilized in applications where structural rectitude and capability to obey the external stimuli is imperative. For micro-fluidics, polymeric structures are utilized as filters for categorization and/or to squeeze out the cells [124]. To guarantee the precise working of an instrument, it is essential that the filter’s structures stay unbroken even at elevated flow rates and the filter does not dislocate itself from the base-plate. Various analytical approaches have been applied to identify the mechanical characteristics of the TPP-ed structures. Interpretation of structural experiments results can be vague since various aspects participate in the mechanical performances of TPP-ed structures [125]. Elevating the laser energy density gives stiffer and higher dimensions voxels. Besides, during fabrication, the voxels’ overlapping changes by changing laser energy density. In this scenario, the fabricated structure becomes stronger with the increment in laser power. The voxels stiffness and overlapping control this development, but it is tough to distinguish their participation and calculate their comparative status. So, the mechanical characteristics of TPP-ed structures force a consideration of the photo-physical process and writing outline used during fabrication.

To determine the mechanical properties, an arrangement named “optical tweezer” was applied to recline a polymeric coil, having a spiral radius equal to 150 nm, which was affixed to a solid polymer anchor on a glass base-plate [126]. The analysis was accomplished with the specimen occupied by an organic solvent. By analyzing the spring retrieval after elongation, the authors concluded the modulus of rigidity (0.5 MPa), which was smaller than the bulk polymer (150 MPa). Two features are the more reasonable reasons to affect the final mechanical properties. However, a thoughtful illustration of these results is not accessible [127]. The primary one is the influence of solvent permeation, which can decline the elasticity [128]. The subsequent one is the polymer glass transition lessening as its magnitudes are contracted to several nano-meters [129]. A difference between the TPP-ed samples mechanical properties and bulk materials was detected while exploring a new and unique analytical technique, though this time, the difference was considered minor. In this situation, atomic force microscopy, equipped with a standardized cantilever, was taken into account to achieve force-distance analyses on polymeric beams manufactured by TPP [128]. The Young’s moduli (*E*) of the manufactured samples were gained using the spring constants and real dimensions. The *E* values were 0.4 GPa and 2 GPa for the TPP-ed structures and bulk polymer, correspondingly. The inconsistency in the *E* values is supposed to be produced by the real fact that TPP-ed structures are usually polymerized on a voxel-after-voxel basis, while the bulk polymers are produced by UV flooding illumination. This study concludes that the comparatively small *E* of TPP-ed structures enables them attractive candidates for soft condensed matter contact measurements.

Meza et al. [130] applied the ideologies of the classified design to prepare structural meta-materials by using: (a) polymers, (b) ceramics, and (c) ceramic + polymer mixtures that were printed in a fractal-like shape. During the in-situ experiments carried out to determine the nano-mechanical properties exposed:

(a) An approximately theoretical grading of mechanical strength and stiffness with comparative density, which outperforms present non-hierarchical nano-lattices.
(b) Recover-ability, with alumina specimens recuperating up to 98% of their initial height after compression up to ≥50% strain.
(c) Suppression of brittle catastrophe and structural uncertainties in ceramic classified nano-lattices.
(d) A variety of distortion mechanisms that can be adjusted by altering the beams’ slenderness ratios.

**Figure 10:**
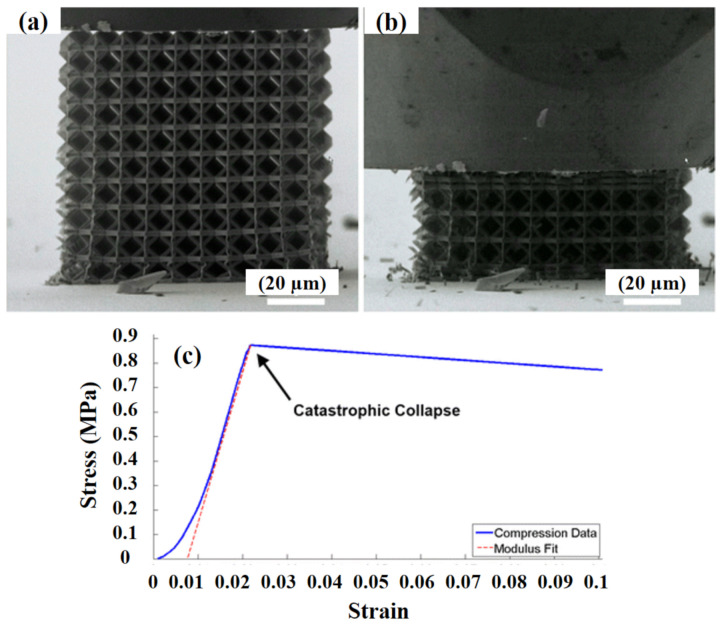
Full structure compression (**a**) starting point of a compression test, (**b**) after failure, and (**c**) stress-strain curve [131]; with permission from Springer.

Supplementary levels of grading beyond a second order did not upsurge the strength and stiffness, suggesting the presence of an optimal degree of grading to intensify resilience. In another study, Meza and Greer [131] reported the production and mechanical distortion of tube lattice structures with characteristics ranging from 10 to 100 nm, hereby denoted as nano-lattices. Titanium nitride (TiN) nano-lattices were prepared using TPP. The structure was made up of octahedral series affixed at their vertices. The uniaxial compression tests in combination with finite element simulations were performed on discrete unit cells. The results exposed that the TiN was capable of enduring tensile stresses up to 1.75 GPa and 1.7 GPa under monotonic and cyclic loadings, respectively, without catastrophe. While carrying out unit cell compression analyses, the beams bi-furcated through oblique torsional buckling, which raises the hyper-plastic behaviour in the load to displacement data. During the compression of the full nano-lattice, the structure distorted at a high strength and modulus that approved well with conventional cellular solid scaling laws, thus giving the low density equal to 1.36%. Figure 10 shows the full nano-lattice compressed at a 250 nm/s displacement proportion until failure occurred (at peak load = 7.50 mN and a displacement = 1.92 lm). The preliminary direct loading was followed by a brittle collapse. The post-deformed image of the structure, presented in Figure 10b, displays the six top-most unit cells compressed until the failure occurred. A complete failure was prohibited due to a restriction imposed by the indenter translating distance. At failure, the stress and strain rate values were 0.873 MPa and 0.0218%, respectively, measured via top-most surface area (=8588 lm^2^) and height (=88 lm). The elastic modulus value was 61.8 MPa, determined via stress to strain loading gradient data.

To increase the material’s strength-weight ratio, one approach is to enhance the strength or decrease the density, or a combination of two can be used. The lightest materials in the solid form have a density value equal to 1000 kg/m^3^. However, the cellular materials such as technical foams can achieve significantly inferior values. However, related to the corresponding bulk materials, their strength is less with an order of 2–3 magnitudes. Cellular topologies can be divided into two types of dominant ways: (a) bending (BDW) and (b) stretching (SDW). Technical foams are usually randomly structured and act as BDW, which is less weight effective, regarding the strength, as compared to SDW. Such behaviour can be observed in regular braced frameworks. Cancellous bone and various other natural cellular solids have an augmented structure. Their base material is organized hierarchically and contains nano-meter sized elements, yielding an advantage in the material’s strength from size effects. Designing cellular materials having an exact micro-architecture can permit one to achieve the structural pros of SDW constructions and size-dependent strengthening effects. Bauer et al. [132] demonstrated that the above-defined materials could be manufactured. By using TPP method, they made and identified the micro-truss and micro-shell structures using alumina + polymer composites. Using size-dependent strengthening in the case of alumina shells was analyzed, mainly when a typical thickness lower than 100 nm was used. The presented cellular materials can attain compressive strengths equal to 280 MPa with densities below 1000 kg/m^3^.

Micro-electromechanical system (MEMS)-based force sensing probes were recently applied to regain the *E* of an acrylic-based resin manufactured using TPP [133]. The test sample was an arrangement of polymeric cubes having 10 µm sides. A layer-after-layer technique was used to manufacture cubes, and a 0.5 µm spacing was used between the parallel lines. The laser scanning speed was 20 µm/s, and the laser power was used in the range of 10 to 23 mW. A MEMS-based force probe was used at the top of the cubes to perform compression testing. Upon the interaction with the polymeric specimen, the probe entered into the sample. The applied force and location were measured, which is the specimen distortion. The sample’s *E* was calculated by using the stiffness and dimensions. In the range of 52 to 185 MPa, a speedy increment in the *E* was detected when the laser power increased from 10 to 23 mW.

#### 3.4.2. Other Properties: Surface Tension, Volume Shrinkage and Optical

There are various types of resins used in TPP. However, multi-functional acrylates have gained significant consideration. It is because the promising characteristics can be achieved by combining acrylic monomers and oligomers [134]. Acrylic molecules are easily available at a low-cost. They have a comparatively steady life and are found in a wide variety of functionalities. In un-polymerized condition, they are soluble in ethanol. Besides several advantages, they show a few restrictions [135]. Particularly, acrylic resins are identified for fabricating in-homogeneous polymeric structures and are subtle to the existence of dissolved oxygen molecules. In TPP, the worst drawback of utilizing acrylic resins is the volume shrinkage (VS) percentage due to volume reduction during the polymerization process [136]. During this process, covalent bonds are formed that reduce the intermolecular distance between monomers. vs. depends on various factors, including the monomers’ molecular structure and the fraction of monomers converted to polymer. vs. can reach up to 20% during the TPP of acrylates and methacrylate. Hence, structures produced by TPP experience a huge magnitude of VS, where stresses develop interfacial defects and substantial distortions. To reduce the VS, different techniques have been used. In one of the techniques, a pre-compensation is utilized throughout the printing of the structure to abandon the deformations during polymerization [137].

In an alternative method, structures have been manufactured at the top of multi- and single-anchor supports to separate them from the base-plate [138]. In this technique, vs. arises in an isotropic way, hence fabricating 3D components having exact dimensions. Besides, new and unique materials that produce 3D structures with a very low vs. have been produced using TPP. One of the materials is zirconia (sol-gel), making organic and inorganic materials with desired features [139,140]. The structures manufactured by this resin showed vs. <1% with high power lasers. In this scenario, micro-fabrication needs a prebaking stage, which involves condensation to generate a solid-film composed of a strong inorganic skeleton. Following on, laser patterning is used to polymerize the film organic section, thus making an unsolvable cross-linked network. TPP is applied to attach pendant methacrylic moieties committed to a solid framework. TPP of this resin generated a material without vs. and deformation. A new unique biochemical technique has been recently demonstrated using thiolene reaction [135]. It was already identified that during UV curing of acrylic and methacrylic substances, the inclusions of multi-functional thiol molecules increase the numerous features of TPP. The capability of thiol molecules to decrease vs. in the polymerization of acrylic resins stems from the observation that the co-polymerization of these monomers advances a cross-linked network with an elevated gel-point conversion. Any vs. ascending before the gel-point development can simply be controlled by viscous flow modifications.

VS can be advantageous in particular applications. For instance, 3D photonic crystals are developed by TPP. Due to vs. isotropy, the entire structure was smaller in size than the analogous fabricated and attached to the substrate. Hence, vs. produced photonic crystals with band gaps moved towards shorter wavelengths [141]. The maximum accuracy in micro-fabrication by TPP is attained when working near-to-threshold environments. The minimum and symmetrical voxels are achieved when laser energy densities are hardly above the essential value to initiate and endure the polymerization process. Inappropriately, these voxels are weak regarding mechanical properties as they are manufactured with a slight polymer conversion degree. As a result, structures show buckling behaviour and break under surface tension while drying. It happens with high aspect ratio, highly complex, or dense structures.

**Figure 11:**
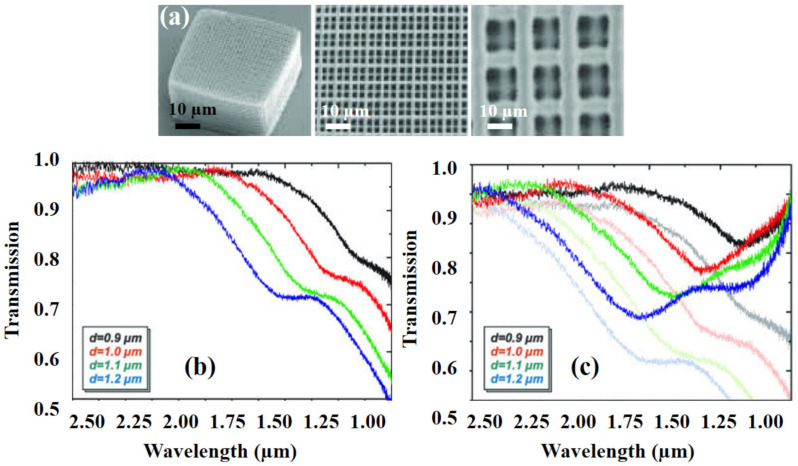
TPP fabricated samples (**a**) SEM images of woodpile structures, (**b**) measured transmission spectra for woodpile structures with different periods without multiplying with Equation (1), and (**c**) measured transmission spectra for woodpile structures with different periods with multiplying with Equation (1) [142]; published under open-access license by OSA publishers.

Serbin et al. [142] reported the 3D printing of woodpile photonic crystal structures manufactured in organic-inorganic polymers and their optical characteristics. Figure 11a shows the SEM image of sich polymers with a thickness equal to 300 nm. The aspect ratio was constantly resulting in FCC symmetric structures. Figure 11b shows the transmission spectra with wavelength. The frequencies of the bandgaps, scaled with the period of the crystals. Hypothetically, the transmittance should rise to unity at frequencies above the bandgap. However, this was not proved in the experiments. To compensate the losses, spectra, as shown in Figure 11c, have been multiplied by a factor:

(1) $$T=T_{measured}{\left(1-0.4\lambda^{-4}\right)}^{-1}.$$

where *T_measured_* is an experimental transmittance, *λ* is the wavelength, and 0.4 is the fitting parameter.

### 3.5. Applications of TPP

#### 3.5.1. Medical

Various TPP applications in medicine have used the fabrication of structures of photocurable polymers and organically modified ceramic (Ormocer) materials. Ormocer materials contain urethane- and thioether (meth)-acrylate alkoxysilanes. The inorganic components are developed from inorganic Si–O–Si networks using hydrolysis and condensation. These networks restrict the material’s VS. In Ormocer materials, the connections between organic and inorganic networks deliver such materials having chemical and thermal steadiness. A range of 3D printed medical devices, including micro-needles, prosthetic device and supports for tissue engineering, can be manufactured using TPP [143,144,145]. The devices, which display lancet, thorn, or hypodermic needle shapes, comprise at least one dimension, less than 1 mm in length. By lessening device magnitudes and diminishing interface with dermal nerve endings, patients’ pain and injury at the injection area may decrease [146]. Solid micro-needles can be used in the same way as traditional transdermal patches. Hollow micro-needles permit diffusion- or pressure-driven transfer of pharmacologic agents via the needle bore to be accustomed over time. An illustration of an Ormocer micro-needle produced by 2PP. Ovsianikov et al. [143] fabricated arrays of in- and out-of-plane hollow micro-needles having 800 μm lengths, base diameters in the range of 150–300 μm and various aspect ratios. Compression load tests confirmed that the micro-needle arrays pierced cadaveric porcine adipose tissue without rupture. Human epidermal keratinocyte viability on the Ormocer surfaces was produced using TPP, same as the control surfaces.

Small bone prostheses are an additional category of medical devices manufactured using TPP. Ovsianikov et al. [147] presented the fabrication of total ossicular replacement prostheses (TORP). TORP produced via conventional materials such as Teflon, Titanium and Ceravital. They confirmed the migration, perforation of the tympanic membrane, trouble in shaping the prostheses and responsiveness with the nearby tissues. An Ormocer prosthesis was implanted and detached from the intended site of use in a frozen human head without impairment. Prosthesis dimensions can be changed per the patient’s anatomy.

#### 3.5.2. Microfluidics

TPP has been used to manufacture novel devices for microfluidics and biomedicine. The lab-on-chip technique needs to be upgraded methods to manufacture micrometer-scaled devices. A vital element in microfluidics is “porous filter,” used in microbiological and bio-chemical analyses [148], which are simple and cost-effective. They are used to isolate plasma from blood cells, to classify different-sized cells. Three-dimensional porous filters manufactured by TPP technology provide advantages in comparison to 2D filters: (a) they have improved mechanical resistance to shear stresses, and (b) they are efficient to filter red blood cells [149]. TPP was used to fabricate 3D porous filters [150]. SZ2080 photoresist was used for the 3D printing of porous filters, made of a zirconium/silicon hybrid sol-gel that gives excellent mechanical steadiness and does not twist during manufacturing. A temperature of 105 °C (90 min) was used to manufacture the sample. The photoresist was hardened, and the solvent was evaporated. The filter was then inscribed by TPP, using 87 MHz recurrence rate, 40 fs pulse period and 800 nm wavelength Ti: Sapphire oscillator, focused with a 1.4 numerical aperture oil immersion microscope objective. The filter was produced in a pentanone shape. Amato et al. [149] reported combining a size-based 3D filter with micrometer-sized pores in a commercial microfluidic chip. The filter was manufactured inside a sealed microfluidic channel using TPP. Tests with a suspension of 3 μm polystyrene spheres in a Rhodamine 6G solution showed that 100% of the spheres were filtered while the fluorescent molecules were conveyed via the filter. The operation was demonstrated for 25 min without any blockage. Schizas et al. [151] employed the TPP to manufacture a readily assembled micro-valve. The valve’s anticipated design was initially assessed, in combination with flow performance, via a computational fluid dynamics study integrating simulated blood pressures and blood veins. The valve design demonstrated a satisfactory performance with nearly 0% blood re-circulation areas being manifest, proving TPP a potential candidate to build medical implants.

#### 3.5.3. Tissue Regeneration

TPP is also a promising candidate for tissue regeneration, drug delivery, and medical sensors. For instance, microneedle arrays for transdermal pharmacologic agents’ delivery, manufactured by ORMOCER, enabled optimal distribution of nanoscale agents to the deep epidermis [102,152] and exert antimicrobial activity after surface functionalization [153,154]. In the last two decades, biophysical factors such as forces have been demonstrated to be potent enough to influence the biological response of cells in culture, even in the absence of biochemical factors [155]. Hence, there is a growing interest in the expansion of biomaterials that mimic mechanical aspects of the cell interaction with its native environment, including the structural role of the extracellular matrix in modulating cell behaviour. The specific structural features of the native cell environment, currently being reproduced in scaffolds, are the mechanical properties [156,157], 2D nano-topography of the cell adhesion substrate [158], and its 3D micro-topology [159]. A primary restriction of existing 3D implantable structures for bone tissue engineering is that a large quantity of cells quickly affixes on the external edges of the structure, limiting the cells diffusion into the internal parts and producing a necrotic core. Besides, these structures usually own an arbitrary arrangement and do not reserve the whole volume isotropy. Paun et al. [160] reported the production and testing of a honeycomb-like structure (HS) that permits in ‘volume’ the relocation of osteoblasts. The probability to regulate the 3D spatial cells growth within these intricate architectures by tuning the free spaces inside the structures was demonstrated. The structures were composed of vertical micro-tubes organized in a multi-layer arrangement, manufactured via TPP of a photopolymer (IP-L780). In-vitro testing confirmed that the cells migration within the 3D structures is due to the partition between the micro-tubes’ layers. Explicitly, for layers partition in the range of 2–10 μm, the cells slowly entered between the micro-tubes. On the other hand, for layer separation <2 μm and >10 μm, the cells could not produce inter-connections and showed deprived mineralization abilities.

## 4. Future Outlook and Conclusions

Recently, direct laser writing (DLW), especially LIFT and TPP, have received substantial consideration. The 3D nature of the LIFT and TPP processes integrated with the length scale of structures permits the manufacturing of functional devices that were impossible to obtain by traditional techniques. Various scientists have added characteristically designed structures to microfluidics, electronics and medical fields. In the near future, the potential application of passive DLW-ed elements can be to develop 3D tissue structures on a chip [161].

There is an excessive curiosity to study the drug interaction with different cells on chips that can simulate an in-vivo atmosphere. For instance, scientists and researchers have developed lung cells at an interface of a twin-folded microfluidic device containing one blood channel and one air channel [162]. Due to the channel compression and extension, the cells behaved as real in a genuine lung and presented excellent results against infections, e.g., pneumonia. LIFT and TPP can benefit by generating 3D channels rather than 2D passive channels, which is essential for next-generation organs-on-chip devices.

In the authors’ opinion, future research should target the manufacturing of active elements based on a larger variety of physical or chemical phenomena. Some DLW-ed microstructures have been utilized as active elements. Besides, magnetically and optically driven micro-rotors have been used in mixers and pumps [163,164]. Other studies have used DLW-ed biomolecules for cell-growth pattern and to develop structures that are able to capture bacteria and practice their flagella [165,166].

LIFT and TPP applications in biomaterials are attracting interest due to their use in the number of applications in biomedicine and biotechnology. Among them, the applications in regenerative medicine are principally attractive, and this field has big opportunities to progress shortly. However, what is particularly relevant about LIFT and TPP developments, is the growing activity in integration at the industrial level. The vital efforts are in progress to determine industrial solutions for PV metallization with LIFT and TPP. An increasing number of startups and well-established companies are involved in developing machine concepts that use LIFT or TPP as the tool of choice for both 2D and 3D DLW of materials.

In 3D printing, a new trend is to apply “topology” optimization. The CAD/CAM user defines the part dimensions, limitations, and the acting forces for the specific software. With these data, the software computes and recommends an optimum shape for the user’s requirements and the maximum confrontation to the forces that will act upon it. The profile presented by the software is, however, most of the time, unconventional and convoluted. Traditional manufacturing methods are often unsuitable for manufacturing it, while 3D printing, in this case, is an appropriate choice for building such parts. The integration of the LIFT or TTP printing method with topology can advance this field.

In summary, we have presented an outline of LIFT and TPP methods for material synthesis at the micro-/nano-level. This review article provides essential material and background to the researchers involved in LIFT and TPP, and motivation to advance the knowledge of the mechanisms tangled in the 3D micro-fabrication by the techniques mentioned above. So far, the advances made in the fields of LIFT and TPP result from the experimental work of various research communities. If the application of LIFT and TPP moves outside of academia and these techniques convert into manufacturing tools, the progress could be exponential. However, there will be a necessity for standardization that can be applied to assess the performance of the apparatus used to carry out LIFT and TPP.

## Figures and Tables

**Table 1:** Various direct laser writing techniques.

Technique	Illustration	References
Selective laser sintering/melting (SLS/SLM)	It involves the melting of powder particles (selective) in a bed of powder via a high laser beam power. A laser heats the powder particles to a point where they are either sintered (SLS) or melt-down (SLM). In these methods, lasers having continuous mode and long pulse are commonly used.	[5,6,7]
Stereolithography (SLA)	It is based on releasing unrestricted radical molecules when the photo-initiator molecules interact with the ultra-violet light. This method is utilized to start the resin polymerization with molecules acting as a precursor and a photo-initiator.	[8]
Laser-induced forward transfer (LIFT)	In LIFT, laser photons trigger the driving force to catapult a minor material volume from a film (source) to a substrate (acceptor). This process is different from the laser-ablation micro-patterning process, where the material is subtracted from the film (source).	[9]
Two-photon polymerization (TPP)	It is an original technique that allows manufacturing any computer-aided designed (CAD) model using a photosensitive polymeric material. This method is flexible and allows the construction of 3D geometry precisely. Using TPP, the scaffold structures’ manufacturing is vital for the cellular processes’ studies, which allow better comprehension of in-vitro tissue development.	[10]

**Table 2:** Various materials printed by LIFT-technique.

Classification of Material	LIFT-Printed Materials	References
Organic materials	Deoxyribonucleic acid	[18]
	Polymers	[19,20]
	Biomaterials	[21,22]
Optical material	Optical structures	[23,24]
Micro-materials	Nanotubes	[25]
	Graphene	[26]
	Particulates	[27,28]
Metallic material	Metals	[29,30]
Gel-type material	Inks	[31,32,33,34]

**Table 3:** Various LIFT methods.

LIFT Methods	Illustration	References
Hydrogen assisted LIFT (HA-LIFT)	It utilizes a laser spot to heat a gas-filled porous layer. After layer heating, the gas temperature elevates; therefore, it explosively expands and destroys the layer locally. It is worthy of mentioning that, in this technique, low energy densities as compared to ablative evaporation are required.	[52,53]
Matrix-assisted pulsed laser evaporation (MAPLE) + LIFT	It is a unique technique that can generate patterns with a variety of materials. It is a combination of LIFT+ MAPLE. It is a pyrolytic method and utilizes in-situ micromachining processes.	[54]
Thermal Imaging (TI)	It uses biological and other materials transmission. In this process, an IR laser spot is split into various small spots, focused via a donor base at a metallic-layer onto which the material has to be coated. The light energy converts into heat and results in gaseous products, and shockwaves are transmitted to the material. Using this technique, one can achieve multi-layers printing.	[55]
Laser-induced thermal imaging (LITI)	It is generally used for polymeric composites with light-emitting properties. In this process, the laser beam operating in continuous mode is concentrated on a film (donor) and placed close to the substrate (receiver). Donor contains (a) translucent substrate, (b) light to heat transformation layer (LHCL), (c) a layer in-between, and (d) a polymeric layer with light-emitting properties. A laser beam is concentrated via the transparent carrier of a donor on the LHCL, and laser beam scanning is done to produce a pattern.	[56]

**Table 4:** Different materials deposited by LIFT with their applications.

Sr. No	Material Transferred	Substrate	Applications	References
01	Lambda phage DNA dissolved in Tris-HCl, EDTA solution	Glass	Research on genome functions	[87]
02	Copper	Quartz	Metal patterns	[88]
03	Polymer composites	Silicon and Gold	Chemical sensors	[89]
04	DNA material and proteins	Poly-L-lysine, nylon-coated glass	Biosensors	[90,91]
05	Fungi (Trichoderma) conidia	Glass	Controlled transfer of organisms	[92]
06	Copper and Aluminum	Mica and Quartz	Contact masks	[93]
07	Blend of LEP and inert polymers (polystyrene)	Poly (3,4-ethylenedioxythiophene) Polystyrene Sulfonate	Organic LED displays	[94]
08	Carbon/binder, LiCoO_2_/carbon/binder	Metal foils	Li-ion micro-batteries and electrodes	[95]
09	Gold, Copper, Nichrome, Barium titanate and Y_3_FeO_12_	Glass, alumina, silicon FR-4 and RO4003 circuit boards	Fabrication of electronics and sensors	[96]
10	p-Si and Al	Silicon	Fabrication of top gated TFTs	[97]
11	Dense oxide phosphor powders of Y_2_O_3_: Eu and Zn_2_SiO_4_: Mn	Alumina, Polymers	Fabrication of high-definition displays	[98]
12	In_2_O_3_	Glass	Microprinted gratings	[99]
13	Au/Sn	Silicon	Die/flip-chip bonding	[100]
14	Pyrene-doped PMMA	PMMA	Patterning of thin film materials	[101]

**Table 5:** Various techniques to enhance the TPP resolution.

Technique	Illustration	References
With the variation of operating conditions	Laser energy density, laser-material interaction time and numerical aperture of the objective are adjusted to achieve high resolution	[119]
Using Radial Quencher	Unlike TPP, the radicals are united with radical quenchers as an alternative of monomers to generate quenched radicals (QRs). These QRs are neutralized by irradiation or heat release. So, the QRs stop the photo-polymerization reactions.	[120]
Designing the high-efficiency photo-initiators	An extremely delicate and effective photo-initiator can participate in a small threshold and quick laser-material interaction time, which would reduce the region’s size where radicals are primarily produced and reduce the radicals’ quantity and dispersal, respectively. Hence, they might be helpful to enhance the TPP resolution.	[121,122]
Simulated emission depletion (STED) like lithography	The contrast between depleting mechanisms and STED is that it includes: (a) two-color photo-initiation, and (b) resolution increment through photo-induced deactivated lithography. Besides, traditional lithography, such as Isopropyl thioxanthone-based depletion lithography, relies on an alternate mechanism for photo-inhibited polymerization, which is absent in STED-lithography	[123]

## Data Availability

Not applicable.
